# Stimuli-disassembling gold nanoclusters for diagnosis of early stage oral cancer by optical coherence tomography

**DOI:** 10.1186/s40580-018-0134-5

**Published:** 2018-01-26

**Authors:** Chang Soo Kim, Dominique Ingato, Petra Wilder-Smith, Zhongping Chen, Young Jik Kwon

**Affiliations:** 1 Department of Chemical Engineering and Materials Science, University of California, Irvine, 916 Engineering Tower, Irvine, CA 92697-2575 USA; 2 University of California, Irvine, Beckman Laser Institute, 1002 Health Sciences Road East, Irvine, CA 92612 USA; 3 Department of Biomedical Engineering, University of California, Irvine, 3120 Natural Sciences II, Irvine, CA 92697-2715 USA; 4 Department of Pharmaceutical Sciences, University of California, Irvine, 147 Bison Modular, Irvine, CA 92697 USA; 5 Department of Molecular Biology and Biochemistry, University of California, Irvine, 3205 McGaugh Hall, Irvine, CA 92697-3900 USA; 6 Department of Chemical Engineering and Materials Science, University of California, Irvine, 1002 Health Sciences Rd, Irvine, CA 92617 USA; 7 Department of Pharmaceutical Sciences, University of California, Irvine, 132 Sprague Hall, Irvine, CA 92697 USA

**Keywords:** Optical contrast agent, Gold nanoparticles, Acid-transforming nanoclusters, Optical coherence tomography, Oral cancer

## Abstract

A key design consideration in developing contrast agents is obtaining distinct, multiple signal changes in diseased tissue. Plasmonic gold nanoparticles (Au NPs) have been developed as contrast agents due to their strong surface plasmon resonance (SPR). This study aims to demonstrate that stimuli-responsive plasmonic Au nanoclusters (Au NCs) can be used as a contrast agent for optical coherence tomography (OCT) in detecting early-stage cancer. Au NPs were clustered via acid-cleavable linkers to synthesize Au NCs that disassemble under mildly acidic conditions into individual Au NPs, simultaneously diminishing SPR effect (quantified by scattering intensity) and increasing Brownian motion (quantified by Doppler variance). The acid-triggered morphological and accompanying optico-physical property changes of the acid-disassembling Au NCs were confirmed by TEM, DLS, UV/Vis, and OCT. Stimuli-responsive Au NCs were applied in a hamster check pouch model carrying early-stage squamous carcinoma tissue. The tissue was visualized by OCT imaging, which showed reduced scattering intensity and increased Doppler variance in the dysplastic tissue. This study demonstrates the promise of diagnosing early-stage cancer using molecularly programmable, inorganic nanomaterial-based contrast agents that are capable of generating multiple, stimuli-triggered diagnostic signals in early-stage cancer.

**Electronic supplementary material:**

The online version of this article (10.1186/s40580-018-0134-5) contains supplementary material, which is available to authorized users.

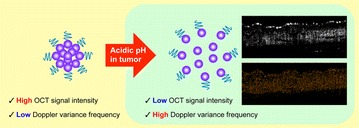

## Introduction

Unique and tunable optical properties [e.g., surface plasmon resonance (SPR) effect] without obstruction by photo-bleaching or photo-blinking have made plasmonic inorganic nanoparticles (NPs) an attractive, popularly-investigated nanomaterial for biomedical imaging applications including optical imaging [1]. Resonant excitation of plasmonic inorganic NPs leads to a large enhancement of the incident electromagnetic field at the NP surface for nonlinear optical spectroscopies such as surface enhanced Raman spectroscopy. [1, 2] One category of plasmonic NPs, gold (Au) NPs do not induce cytokine secretion, making them highly biocompatible and applicable to a number of delivery, sensing, and imaging applications [3–6]. Au NPs have been most intensively investigated for imaging applications over the last few decades; well-established methods of Au NP synthesis and surface modifications have allowed for control over their morphology-dependent optical properties [7–13]. Among many imaging applications, optical coherence tomography (OCT) is a fast, non-invasive, and high resolution imaging modality that uses a Michelson’s interferometer with a low coherence light source [14]. OCT’s two- and three-dimensional high resolution imaging capability is well-suited for detecting specific stages of diseases such as cancer [15–18]. However, the challenge of obtaining disease-specific molecular contrast undermines the many promising features of OCT [19–22]. This limitation particularly affects OCT diagnostic performance for early-stage cancer; however, it can potentially be overcome by using optical contrast agents such as Au NPs [3, 23–26]. For example, significantly enhanced OCT contrast in vivo was demonstrated by administering anti-epidermal growth factor receptor-conjugated Au NPs via a multimodal delivery method to neoplastic tissues [27].

Plasmonic Au nanoclusters (NCs) that transform their physical and optical properties upon detecting a stimulus (e.g., mildly acidic pH of tumor tissue [28–30]) in a diseased area are highly promising OCT contrast agents due to their ability to improve OCT imaging with enhanced SPR effects [31, 32]. Moreover, pH-transforming systems of Au NPs have previously been described in the literature; one approach includes Au NPs composited in pH-responsive polymers that expand under acidic conditions due to changes in electrostatic forces [33, 34]. In this study, we synthesized Au NCs by using an acid-cleavable linker to cluster individual Au NPs.

**Fig. 1:**
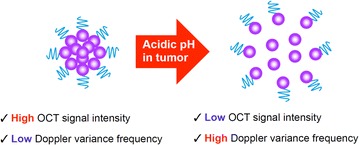
Schematic illustration of OCT signal changes upon acid-triggered transformation of Au NCs. Small Au NPs (purple) are clustered via acid-cleavable linkers and further tethered with polyethylene glycol (PEG; blue) to prevent serum-mediated aggregation. Under acidic conditions (tumor microenvironment), the individual Au NPs will be dispersed, generating significantly reduced scattering intensity and increased Doppler variance frequency

We hypothesized that the mildly-acidic tumor microenvironment would disassemble acid-degradable Au NCs to individual Au NPs, resulting in OCT’s Doppler variance frequency shift due to increased Brownian motion as well as decreased intensity signals upon decreasing SPR effect. Acid-degradable Au NPs were clustered via acid-cleavable linkers to pinpoint mildly acidic tumor tissue [28, 30]. Spectral domain (SD) and Doppler variance (Dv) OCT modes were used to characterize morphological changes from clusters (scattering-dominant with SPR effect; slow Brownian motion) to dispersed Au NPs (absorption-dominant with SPR effect; fast Brownian motion) (Fig. 1). The Au NCs generate high scattering signal in normal tissue due to strong inter-particle SPR effect while substantially reducing scattering signal in mildly acidic tumor tissue upon disassembly to small individual Au NPs. Simultaneously, the disassembled Au NPs in tumor tissue increases the Doppler variance frequency spectrum due to their small size (increased Brownian motion), which can be quantified by DvOCT. [35] Therefore, the stimuli-triggered disassembly of Au NCs can be used to visualize a neoplastic site with high resolution by multiple optical signal changes: the loss of scattering signal and an increase in Doppler variance frequency spectrum [36, 40]. In vivo OCT imaging pinpointed the site of early-stage oral dysplasia via acid-disassembly of Au NCs and validated the hypothesis of this study.

## Methods/experimental

### Materials

Hydrogen tetrachloroaurate (III) trihydrate (HAuCl_4_·3H_2_O) and trisodium citrate dihydrate (Na_3_C_6_H_5_O_7_·2H_2_O) were purchased from Sigma Aldrich (Milwaukee, WI). *N*-Succinimidyl 3-(2-pyridyldithio)-propionate (SPDP) and amino PEG monomethyl ether (2 kDa) were purchased from Pierce (Rockford, IL). 2-Aminoethanol, pyridinium *p*-toluenesulfonate, ethyl trifluoroacetate, and 2-methoxypropene were supplied by Acros (Morris Plains, NJ). Dimethly sulfoxide (DMSO), sodium azide, and bovine serum albumin were purchased from Fisher Scientific (Fair Lawn, NJ). Dithiothreitol (DTT) was supplied by Fisher Scientific (Pittsburgh, PA) and diethylene glycol bis (3-amino propyl) ether was obtained from TCI (Tokyo, Japan). Carboxy-SNARF-1 (SNARF) was purchased from Invitrogen AB (Frolunda, Sweden). A carbon-coated copper TEM grid was purchased from Electron Microscopy Sciences (Hatfield, PA).

### Synthesis of Au NPs

Citrate-capped Au NPs (15 nm in diameter) were synthesized by following a previously reported protocol with slight modifications [37]. Briefly, 100 milliliters of 1% (w/w) HAuCl_4_·3H_2_O in Milli-Q water (18.2 MΩ) was heated to its boiling point (100 °C) and added dropwise with 4 mL of 1% (w/v) Na_3_C_6_H_5_O_7_·2H_2_O in Milli-Q water (18.2 MΩ) with vigorous stirring. Upon addition of Na_3_C_6_H_5_O_7_·2H_2_O, the color of the mixture changed to deep red instantaneously. After reflux for an additional 15 min, the resulting solution was cooled down to room temperature with continuous stirring. After overnight incubation at 25 °C, the resulting Au NPs were characterized by using a Phillips CM 20 transmission electron microscope (TEM) (Philips Electronic Instruments, Mahwah, NJ) and ZEN3600 Zetasizer dynamic light scattering (DLS) particle analyzer (Malvern Instruments, Worcestershire, UK). It was shown that Au NPS were 15 nm (± 0.3 nm) in diameter and spherical in shape (TEM). The concentration of the resulting Au NPs was calculated to be 3.44 × 10^12^ particles/mL using a previously described UV/Vis spectroscopic method [38]. All glassware used in the Au NP synthesis was cleansed in aqua regia, 3:1 (v/v) HCl:HNO_3_, and thoroughly rinsed with deionized (DI) water.

### SPDP-activation of Au NPs

Ten milliliters of Au NPs (3.44 × 10^12^ particles/mL) were washed with Milli-Q water (18.2 MΩ) by repeated centrifugation at 10,000 rpm for 30 min and then re-dispersed in Milli-Q water (18.2 MΩ). After the 2 times wash steps, Au NPs were re-dispersed in 10 mL of 0.02 M sodium bicarbonate buffer (pH 8.73). Using a peristatic pump, ten milliliters of Au NPs (3.4 × 10^12^ particles/mL) in sodium bicarbonate buffer were steadily added at 7 μL/s to 2 μL of 20 mM SPDP solution in dimethyl sulfoxide (DMSO) (4.73 × 10^−20^ mol SPDP per Au NP) while the solution was stirred vigorously on ice. The reaction was left stirring at room temperature overnight. Unreacted SPDP was removed by centrifugation twice at 10,000 rpm for 30 min, and SPDP-activated Au NPs were re-dispersed in 10 mL of 0.02 M sodium bicarbonate buffer and briefly sonicated in a sonication bath for 30 s [39]. When exposed to dithiothreitol (DTT), SPDP cleaves; this concept was used to quantify SPDP conjugation. SPDP conjugation of Au NPs was quantified by the release of a pyridine-2-thione group when incubated with dithiothreitol (DTT). Briefly, 1.5 mL of SPDP-activated Au NP solution (3.4 × 10^12^ particles/mL) was washed twice with DI water by repeated centrifugation at 10,000 rpm for 30 min and re-dispersion. Seventy-five microliters of 15 mg/mL DTT in DI water were added to 0.75 mL of SPDP-Au NP solution, followed by incubation for 15 min at room temperature. The released amount of pyridine-2-thione was quantified by the absorbance at 343 nm using a Varian Cary 50 UV/Vis spectrophotometer (Varian Inc., Palo Alto, CA). After subtracting the background absorbance of a DTT-free SPDP-Au NP solution at the same concentration, SPDP-conjugation was calculated to be 5.34 × 10^−22^ mol (= 321 mol) of SPDP per Au NP. The resulting SPDP-activated Au NPs were kept at 4 °C without exposure to light.

### Au NC synthesis and characterization

Seven hundred fifty microliters of SPDP-activated Au NPs (6.8 × 10^12^ particles/mL) were added slowly into acid-cleavable diaminoketal (DAK) solution in 0.02 M sodium bicarbonate solution on ice with strong stirring. Then, the mixture remained at room temperature overnight with continuous stirring. The unreacted cross-linkers were washed away by centrifugation at 8000 rpm for 10 min and re-dispersed in 0.02 M sodium bicarbonate solution. DAK conjugated Au NPs were characterized by dynamic light scattering (DLS) and UV/vis spectrometer. 100 μL of DAK conjugated Au NPs were slowly added to 10 mL of SPDP-activated Au NPs on ice, and the mixture was kept stirring overnight. The final Au clusters were added to 160 μL of amine-functionalized PEG (MW = 2 k Da, 10 mg/mL) with strong stirring. The acid degradable Au clusters were separated from free, SPDP-activated Au NPs by centrifugation at 6000 rpm for 30 min [35]. The non-acid degradable (control) Au clusters were synthesized by the same method except diethylene glycol bis (3-amino propyl) ether (0.05 g/mL) was used instead of DAK. The sizes of the Au NPs and Au NCs were determined using a Phillips CM 20 transmission electron microscope (TEM) (Philips Electronic Instruments, Mahwah, NJ) and ZEN3600 Zetasizer dynamic light scattering (DLS) particle analyzer (Malvern Instruments, Worcestershire, UK). The absorbance of Au NCs was measured using a Varian Cary 50 UV/Vis Spectrophotometer (Varian Inc., Palo Alto, CA).

### OCT/DvOCT imaging configuration

Spectral-domain optical coherence tomography (SD-OCT) [27] was used to image the scattering and Doppler variance of the acid-degradable and non-acid degradable Au NCs in droplets and hamster cheek pouch tissues. Doppler variance OCT images (DvOCT) were also obtained based on the power spectrum of the temporal fluctuations of the OCT magnitude using the SD-OCT system. Low-coherence light with a 1310 nm center wavelength and 90 nm full width at half maximum (FWHM) was used, and imaging depth and depth resolution were 3.4 mm and 8 µm in air, respectively. A 2-axis scanner with two galvanometers was located at the same sample arm. All SD-OCT and DvOCT images were obtained with the same focal point. A 130 nm wide spectrum was sampled by a 1 × 10^24^ InGaAs detector array at a 7.7 kHz frame rate. Acid degradable Au-NCs (1.62 × 10^9^ particles/mL) were mixed with pH 5 solution in 37 °C. Then, SD-OCT and DvOCT images were obtained every 30 min with 3 µL of aliquot solution for 2 h.

### OCT/DvOCT imaging of Au NCs incubated in DI water and pH 5.0 acetate buffer

The acid-degradable and control Au NCs (960 μL, 7.05 × 10^9^ particles/mL) were concentrated to 10 μL of solution by centrifugation at 2000 *g* for 30 min. The resulting solution (3 μL) was mixed with 6 μL of DI water as a control, and 7 μL of the solution was mixed with 14 μL of pH 5.0 acetate buffer to hydrolyze the Au clusters. After 2 h of incubation at 37 °C, a 3 μL aliquot of each sample was dropped on the polyethylene substrate, and OCT/DvOCT images were obtained. The droplet OCT/DvOCT images with different pH conditions were quantified with the Scion Image Process (Scion Corporation).

### SNARF-conjugation on Au clusters

Au NP clusters were rinsed with Milli-Q water (18.2 MΩ) by repeated centrifugation at 6000 rpm for 30 min and re-dispersion in Milli-Q water (18.2 MΩ). In order to functionalize with amino groups, 1.5 mL Au NP clusters (7.05 × 10^9^ particles/mL) in Milli-Q water (18.2 MΩ) were then reacted on ice with vigorous stirring with 3.75 μL of 0.05 g/mL diethylene glycol bis (3-amino propyl) ether in Milli-Q water (18.2 MΩ) (molecular ratio of diethylene glycol bis (3-amino propyl) ether to SPDP is 1.4 x 10^5^). After removing unreacted diethylene glycol bis (3-amino propyl) ether by two centrifugation steps (6000 rpm for 30 min), 1.5 mL of Au NP clusters (7.1 × 10^9^ particles/mL) re-dispersed in Milli-Q water (18.2 MΩ) were added to 0.05 g/mL SNARF succinimidyl ester in Milli-Q water (18.2 MΩ) on ice, followed by stirring at room temperature overnight without exposure to light. SNARF-conjugated Au NP clusters were obtained in Milli-Q water (18.2 MΩ) after removing un-conjugated dyes by centrifugation at 6000 rpm for 30 min. DLS, UV/vis spectrometer, and TEM were used for the characterization after the SNARF-conjugation.

### In vivo OCT imaging

To establish the oral cancer model, golden Syrian hamsters (*Mesocricetus auratus*, Harlan Sprague–Dawley, San Diego, CA) were topically treated with 0.5% (w/v) 9, 10-dimethyl-1,2-benzanthracene (DMBA, Sigma) in mineral oil three times per week for 4-6 weeks to induce dysplasia in one cheek pouch in each animal. The contralateral cheek pouch of the hamster received mineral oil application only. All animal protocols were approved by the Institutional Animal Care and Use Committee (IACUC, 97-1972) at the University of California, Irvine. Acid degradable and control Au clusters were synthesized, characterized, and applied on the hamster cheek pouches using a multi-modal delivery method (microneedles for penetration and ultrasound for distribution of Au NCs). OCT/DvOCT was used to observe the optical property changes of stimuli-responsive Au clusters. All the OCT/DvOCT Imaging was performed in the cheek pouch of the anesthetized hamster by gently clamping the tissues to a microscope stage using a custom-built, ring-shaped clamp. CR3 roller microneedles (MTS dermaroller with 200 μm depth; Clinical Resolution Laboratory, Inc., Beverly Hills, CA) were rolled on both the DMBA-untreated and control sides of the hamster cheek pouches three times at three different angles. Then, 200 μL of Au cluster solution (7.05 × 10^9^ particles/mL) were administration by dropping it directly into the 1-cm-diameter aperture of the ring-shaped clamp. After 10 min Au cluster topical administration, ultrasonic force (0.3 W/cm^2^ of 1 MHz) was applied to the cheek pouch using the Dynatron 125 ultrasonicator (Dynatronics Corporation, Salt Lake City, UT) for 1 min in the presence of ultrasound gel, followed by OCT/DvOCT imaging. After OCT/DvOCT imaging, all the OCT/DvOCT images were quantified with the scion image process (Scion Corporation).

## Results and discussion

### Synthesis and characterization of acid-disassembling Au NCs

In order to meet the design goals of this study, Au NCs needed to be composed of small Au NPs of the minimally required size for detection by OCT and DvOCT. Although higher OCT/DvOCT signals can be obtained using large Au NPs [41], the resulting Au NCs should be small enough (e.g., less than 100 nm [42]) to penetrate and readily disperse in target tissues. In a previous study, we demonstrated that ~ 70 nm Au NPs could be detected at the site of early-stage oral dysplasia in vivo after topical delivery by combined microneedle (penetration through stratum cornea) and ultrasound (dispersion in oral dysplasia) delivery [27]. Therefore, the design goal of this study was to create Au NCs that were smaller than 100 nm and able to disassemble into individual 15 nm Au NPs under mildly acidic conditions.

**Fig. 2:**
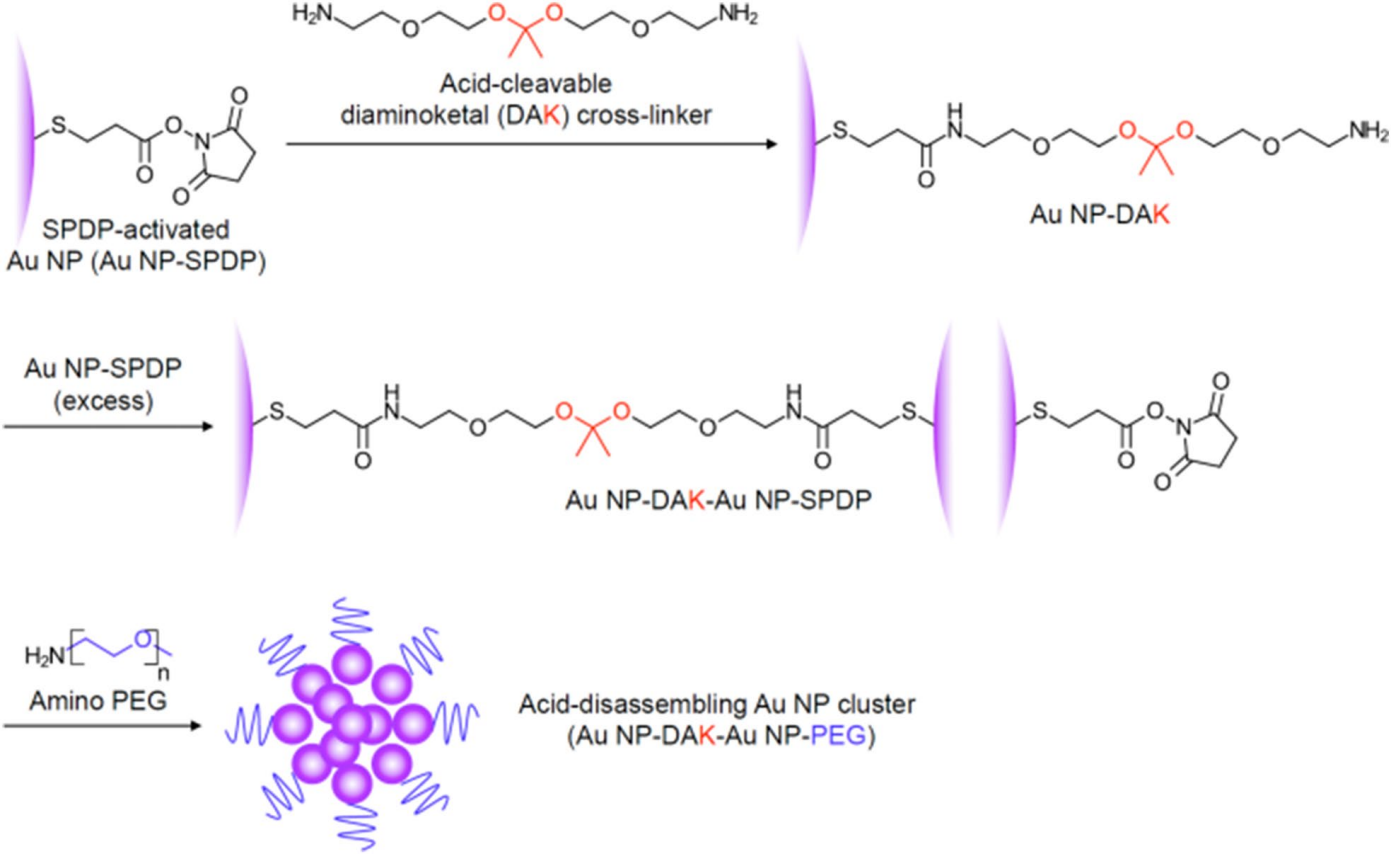
Synthesis of acid-degradable Au NCs. SPDP-activated Au NPs were conjugated with acid-cleavable diaminoketal (DAK) cross-linkers. DAK cross-linker-conjugated Au NPs were then mixed with excess SPDP-activated Au NPs to form clusters. The resulting Au NCs were PEGylated by reaction of amino PEG with the NHS group available on the surface of SPDP-activated Au NCs

Briefly, 15 nm Au NPs were synthesized using the same method that was previously reported with slight modifications [27]. Then, the thiol groups on the NP surface were conjugated with *N*-succinimidyl 3-(2-pyridyldithio)-propionate (SPDP), a heterofunctional cross-linker with amine-reactive NHS and thiol-reactive pyridyldithiol groups, generating Au NPs with NHS groups on their surface (Au NP-SPDP). Addition of excess acid-cleavable ketal cross-linker (diaminoketal [DAK]) to the Au NP-SPDP, followed by removal of unreacted DAK, resulted in Au NPs with amine groups conjugated on the surface (Au NP-DAK). The DAK cross-linker was synthesized as previously reported [43]. The amine groups on the surface of Au NP-DAK were then used to anchor Au NP-SPDP via their surface NHS groups, resulting in Au NP-DAKs clustered by Au NP-SPDP (Au NP-DAK-Au NP-SPDP). Finally, the remaining NHS groups on the surface of the surrounding Au NPs were further conjugated with polyethylene glycol (PEG) with an amine group. PEG was conjugated on the surface of Au NCs to prevent aggregation, particularly under biological/physiological conditions [44]. The synthesis of acid-disassembling Au NCs is illustrated in Fig. 2. Non-acid-degradable (control) Au NCs were also synthesized by the same method except diamino cross-linker without a ketal linkage. More specifically, diethylene glycol bis (3-amino propyl) ether (0.05 g/mL) were used instead of DAK. Au NP size, the ratio of Au NP-SPDP to Au NP-DAK, and the molecular weight of PEG control the size of the resulting Au NCs.

### Acid-responsive transformation of Au NCs and optical signal changes

**Fig. 3:**
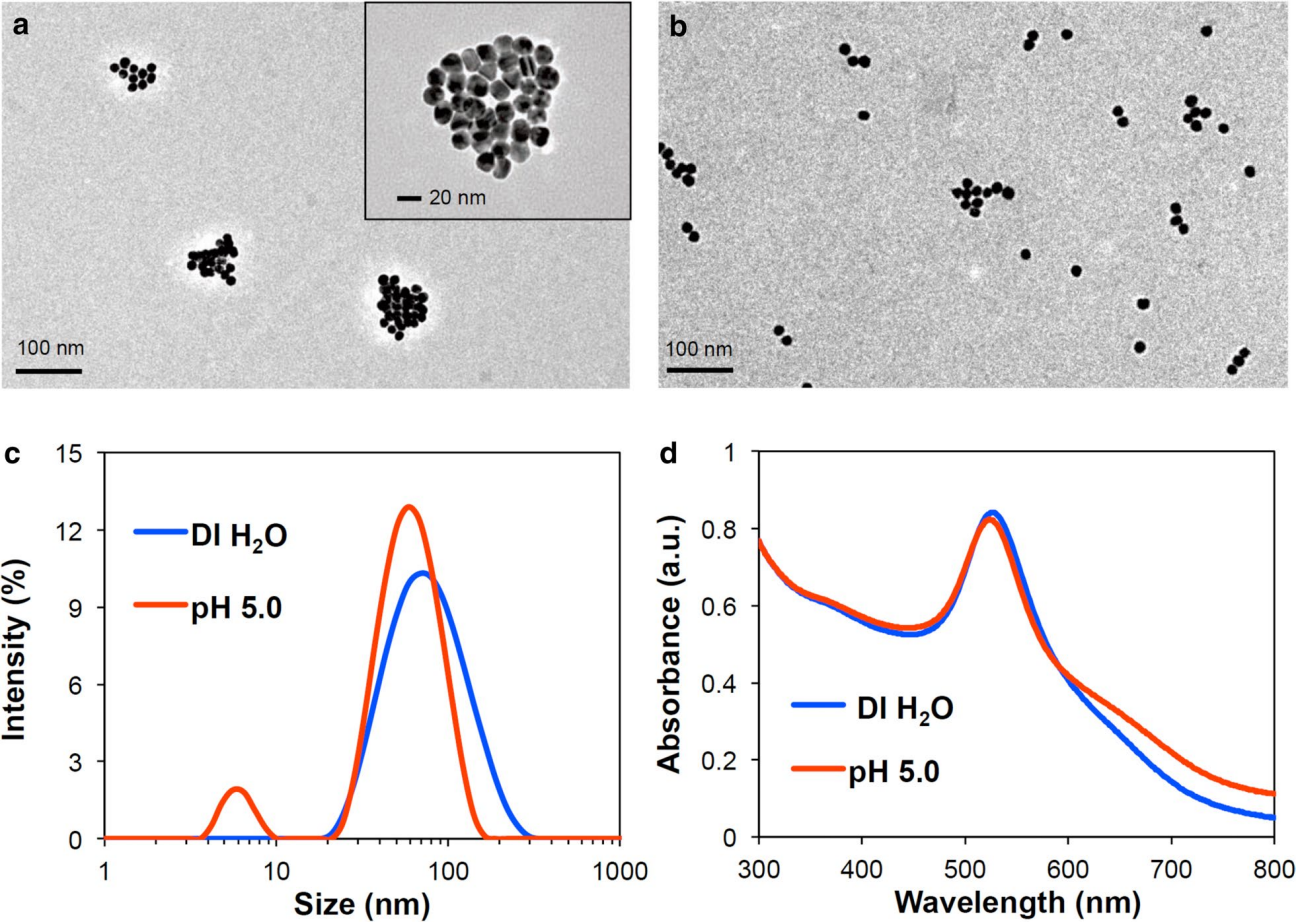
Characterization of Au NCs. Acid-responsive changes in size and morphology of Au NCs incubated in DI water and 100 mM acetate buffer (pH 5.0) at 37 °C for 2 h, confirmed by TEM (**a** DI water; **b** pH 5.0), DLS (**c**), and UV–Vis spectra (**d**)

As intended, the resulting acid-disassembling Au NCs were approximately 85 nm in diameter, while non-acid-disassembling Au NCs were 80 nm. The sizes of particles were determined by dynamic light scattering (DLS), followed by transmission electron microscopy (TEM). In this proof-of-concept experiment, acid-responsive disassembly of the resulting Au NCs was investigated by incubating them in DI water and an acetate buffer (100 mM; pH 5.0), a conventionally used acidic condition. As shown in TEM images (Fig. 3a, b), acid-responsive Au NCs rapidly disassembled at pH 5.0. The acid-disassembly of Au NCs from covalently clustered Au NPs in DI water (Fig. 3a) to individual Au NPs with slight aggregation at pH 5.0 (Fig. 3b) was also indicated by appearance of split size peaks in DLS measurement (91% at 64 nm and 9% at 6 nm in intensity) (Fig. 3c). Simultaneously, the SPR absorption peak was shifted from 531 to 527 nm (Fig. 3d). As shown in Fig. 3b, there were only slightly aggregated Au NPs (40–80 nm) and individual Au NPs (15 nm). Therefore, the peak for small particles (6 nm) (Fig. 3c) could be an artifact in DLS measurement. DLS determines particle sizes based on lumped Brownian motion, rather than tuning for individual sizes. Therefore, signal intensity generated by larger particles is greatly weighted over those generated by smaller ones. Taken together, the results shown in Fig. 3 confirmed the acid-triggered morphological changes of Au NCs. pH 5.0 was used in this study as a commonly used acidic condition in many reports. The disassembling kinetics of Au NCs would be affected by different pHs, resulting in faster or slower transformation.

**Fig. 4:**
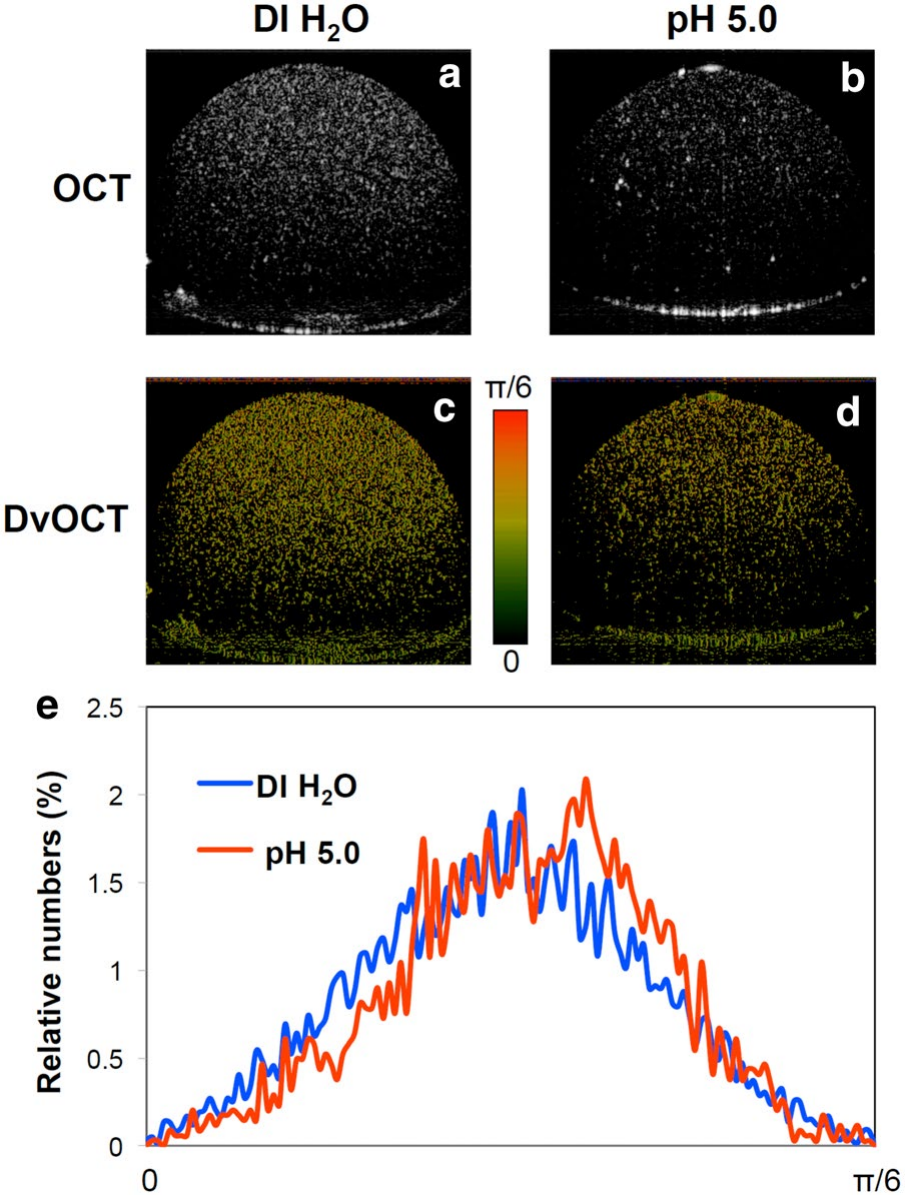
Acid-disassembly of Au NCs observed by OCT and DvOCT. Images of acid-disassembling Au NCs in droplets (3 μL) obtained using SD-OCT (**a**, **b**) and DvOCT (**c**, **d**) after incubation in DI water or pH 5.0 acetate buffer at 37°C for 2 h. Fast and slow moving particles (**c**, **d**) were color-coded with red and green, respectively, and further quantitatively analyzed (**e**)

OCT imaging was explored with stimuli-responsive Au NCs to detect distinct signal changes including scattering intensity and Doppler variance. Acid-disassembling Au NCs were incubated in DI water and pH 5.0 at 37°C for 2 h, as in Fig. 4. Then Au NC-containing droplets (3 μL) were imaged using spectral domain (SD)-OCT and DvOCT at the same focal points (Fig. 4). The high SPR effect associated with closely packed plasmonic Au NPs caused acid-disassembling Au NCs in DI water (Fig. 4a) to generate substantially stronger scattering signals than the sample in pH 5.0 buffer (Fig. 4b). For DvOCT, acid-disassembling Au NCs in pH 5.0 buffer (Fig. 4d) showed greater Doppler variance signal than the sample in DI water (Fig. 4c; the appearance of fast moving particles [colored red]) upon disassembly of relatively large Au NCs to smaller, more mobile Au NPs. Disassembled individual Au NPs (15 nm) have the absorption dominant optical property, compared to assembled Au NPs (85 nm). Doppler variance of all pixels in DvOCT images was quantified. The quantitative plot (Fig. 4e) showed an obvious increase in Brownian motion brought about by Au NC disassembly at pH 5.0 as compared to in DI water. Taken together, proof-of-concept experiments (Figs. 3, 4) clearly demonstrated acid-triggered disassembly of Au NCs generates distinct optical signal changes in OCT and DvOCT images.

### Confirmation of mildly acidic pH in early-stage oral dysplasia

**Fig. 5:**
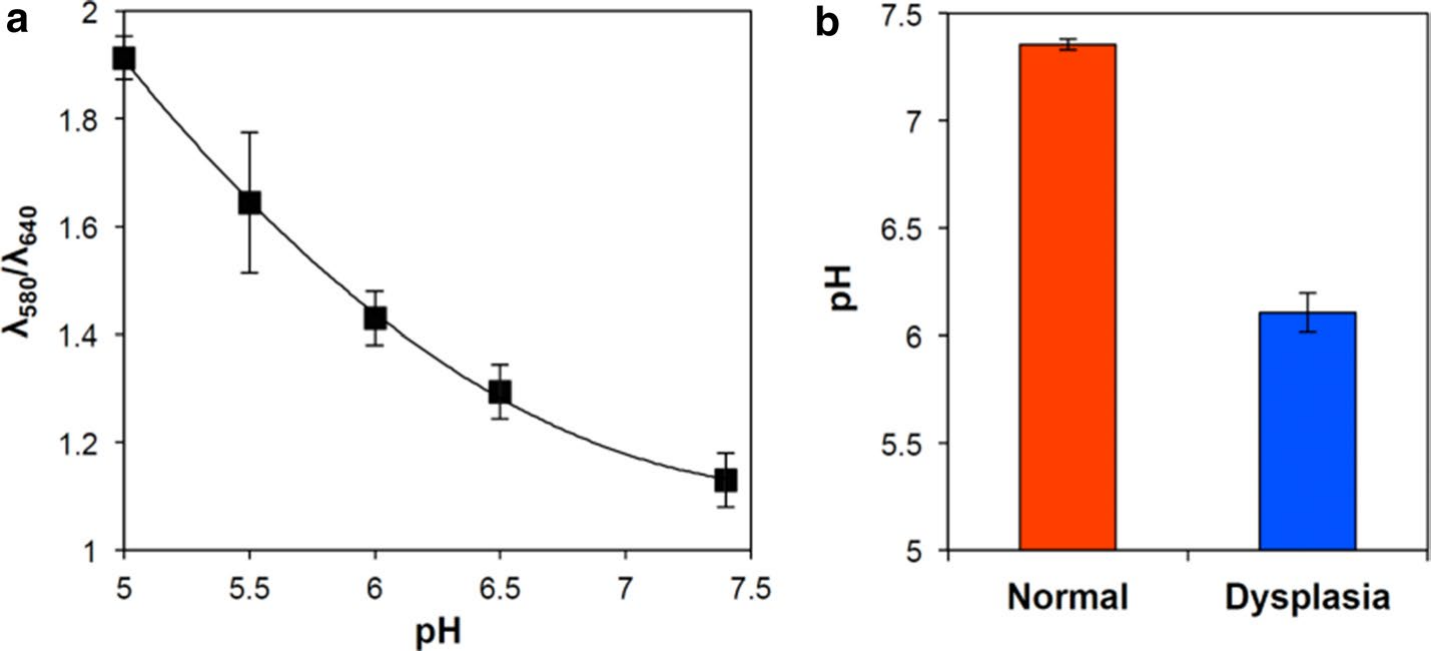
Characterization of pH in normal and dyplastic tissue. pH-dependent fluorescence ratios of Au NC-SNARF at 580 and 640 nm wavelengths (λ _580 nm_/λ _640 nm_) at various pHs (standard curve) (**a**) and pHs in normal (applied with mineral oil alone) and oral dysplastic (applied with DMBA in mineral oil) tissues, determined by topically applying Au NC-SNARF using multi-modal delivery methods and comparing resultant fluorescence ratios by confocal microscopy at 580–640 nm wavelengths in the tissues using confocal microscopy (**b**)

Low pH condition, especially, pH 5.0, is a commonly employed stimulus in acid-responsive intracellular drug delivery as endosome-mimicking condition [45]. However, real extracellular pH in tumor tissue contributed to by many different molecular and physiological changes during carcinogenesis [46, 47] greatly varies in the range of pH 6.5–7.2. Therefore, it was necessary to confirm the pH of early oral dysplasia. In this study, a hamster cheek pouch model was used as an early-stage oral dysplasia model because it has a variety of common features with human oral cancer including neovascularization during carcinogenesis, oncogenic expression, and immune response [48]. A proven carcinogen (9,10-dimethyl-1,2-benzanthracene (DMBA)) was used to induce squamous neoplasia. To measure the pH of the tissue from the animal model, a pH-sensitive fluorescent dye, carboxyl SNARF, was conjugated (Au NC-SNARF) to the Au NC surface following the general method described in Fig. 1. SNARF on Au NC surface generates pH-dependent fluorescent emission ratios at 580 nm to 640 wavelengths upon excitation by a 488 nm wavelength light [49]. Before beginning animal experiments, the fluorescence of Au NC-SNARF at 580 nm vs. 640 nm emission wavelengths in varying pH 5.0–7.5 buffers was compared to obtain a standard curve (Fig. 5a). Then Au NC-SNARFs were topically administered on the DMBA-treated and control (mineral oil-treated) hamster cheek pouches using the combined microneedle and ultrasound delivery method as was reported previously [27]. Confocal images of the DMBA-treated cheek pouch and control cheek pouch were taken at 580 nm and 640 nm emission wavelengths with excitation at 488 nm wavelength. Then images in 50 by 50 pixels were obtained and processed to quantify fluorescence intensities at 580 nm vs. 640 nm. As shown in Fig. 5b and Additional file 1: Figure S1, the pH in the DMBA-applied cheek pouch tissue was determined to be mildly acidic (approximately 6.2), while the pH in the mineral oil-applied cheek pouch tissue was found to be close to a normal physiological condition (approximately 7.3).

**Fig. 6:**
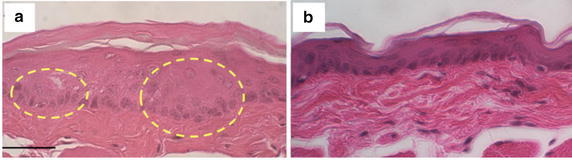
H&E stained dysplastic hamster cheek pouch tissue topically administered with acid-disassembling Au NCs (**a**) and normal hamster cheek pouch tissue (**b**). Dotted circles indicate thickened epithelial layers indicating early-stage dysplasia. Scale bar: 100 μm

After the fluorescence imaging in vivo, the oral tissues were collected and stained with hematoxylin and eosin (H&E) to histopathologically confirm early-stage oral dysplasia in the DMBA-applied cheek pouch compared to normal tissue (Additional file 1: Figures S2 and Fig. 6). The image of the DMBA-applied tissue showed thickened epithelial layers and early-stage epithelial down growth underneath of the stratum cornea (circled area in Fig. 6a), indicating an early-stage dysplastic event, while the epithelial layers remained unchanged in the contralateral cheek pouch (Fig. 6b).

In addition, no adverse effects (e.g., inflammation) from the Au NCs were observed in either cheek pouch tissue sample, implying high biocompatibility of the administration methods. The results shown in Figs. 5 and 6 confirm that mild acidification (pH 6.2) is indeed a promising pathological trigger in early-stage oral dysplasia for Au NC disassembly. We previously reported that the ketal linkage used in synthesizing Au NCs in this study readily hydrolyzes not only at pH 5.0 but also at pH 6.0 in cell culture [43]. Therefore, acid-triggered Au NC disassembly and simultaneous generation of optical signal changes are anticipated in early-stage oral dysplasia (pH 6.2).

### Pinpointed, multi-modal, optical diagnosis of early-stage oral cancer using acid-disassembling Au NCs

**Fig. 7:**
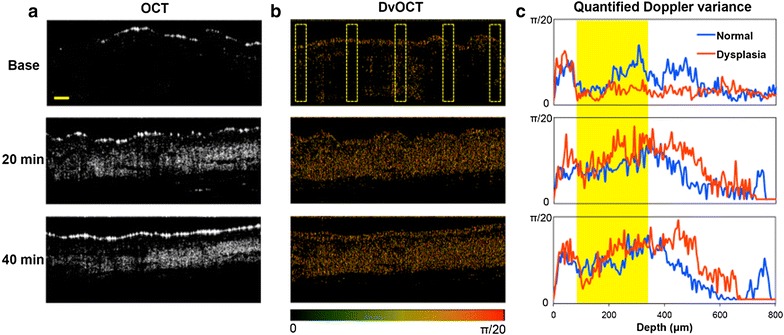
OCT and DvOCT imaging of dysplastic hamster check pouch with acid-disassembling Au NCs. Images of DMBA-applied, early-stage dysplastic hamster cheek pouch, obtained by SD-OCT (**a**) and DvOCT (**b**) after administering acid-disassembling Au NCs. Scale bar: 100 µm. The images were taken before (base) and 20 and 40 min after administering acid-disassembling Au NCs. The color-coded Doppler variance in DvOCT images of early-stage dysplasia (5 dotted regions) was quantified and compared with that of normal tissue (**c**) (yellow area: epithelial layers)

Acid-disassembling Au NCs were topically administered on the DMBA-applied hamster cheek pouch (oral dysplasia) and the other control cheek pouch (normal) as described earlier. In order to minimize OCT signal changes resulting from measurement errors such as tissue movement and different scattering coefficient of skin at different imaging depth, [50] a constant focal point of imaging in the cheek pouch of the anesthetized hamsters was maintained. Both scattering and Doppler variance images were obtained using SD-OCT and DvOCT, respectively, at different time points after Au NC administration (Fig. 7).

**Fig. 8:**
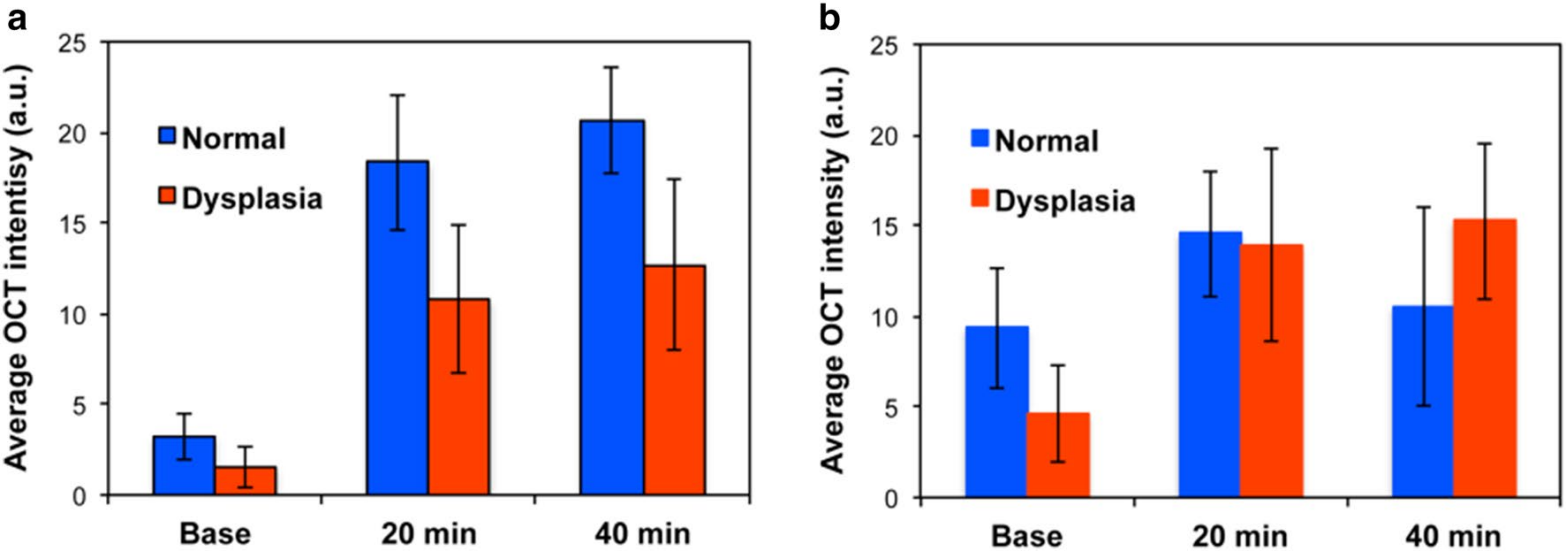
Quantification of OCT results. Average SD-OCT intensity for **a** acid-degradable Au–NCs and **b** non-acid degradable Au-NCs after before (base) and after multi-modal delivery method (20 and 40 min) in 5 different dotted SD-OCT image regions in Figs. 7 and Additional file 1: Figure S3

First of all, very minimal scattering optical signals were obtained before administering Au NCs (base in Figs. 7a, 8). The Doppler variance in epithelial layers in the oral dysplasia tissue without Au NCs (base) was lower than that in normal tissue (Fig. 7b, c), possibly due to the tight junctions between the overgrown epithelial cells (Fig. 6a). However, when acid-disassembling Au NCs were administered, significantly improved scattering images (Figs. 7a and 8) and Doppler variance images (Fig. 7b) were obtained from dysplastic tissue and from normal tissue (Additional file 1: Figures S3 and S4). OCT images clearly showed irregular stratification of the epithelial layers beneath the stratum cornea (bright top line) in oral dysplasia (Fig. 7a), while normal stratification of the epithelial layers was found in control tissue (Additional file 1: Figures S3 and S4). As hypothesized, lower scattering intensity was obtained in the oral dysplastic tissue than in normal tissue (Figs. 7, 8). Administration of acid-disassembling Au NCs also significantly improved DvOCT images (Additional file 1: Figures S3; Figs. 7b, and 8). Most notably, the Doppler variance in the epithelial layers (yellow area) substantially increased in the dysplasia (increased red pixels as compared to the base case in Fig. 7b). The epithelial layers are the sites of mild acidity as caused by localized early-stage dysplasia (Figs. 5, 6). Also, Doppler variance became even higher in the dysplastic tissue than that in the normal tissue (Additional file 1: Figures S3 and Fig. 7c). Therefore, the results shown in Fig. 7 demonstrated acid-triggered disassembly of Au NCs to Au NPs by increased Doppler variance (Brownian motion) in the epithelial layers in the dysplastic tissue.

**Fig. 9:**
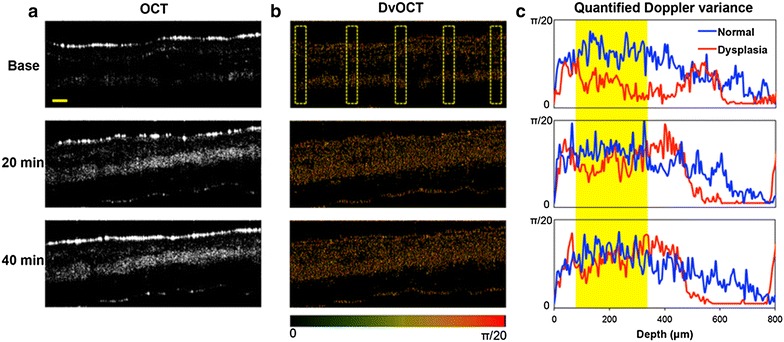
OCT and DvOCT imaging of dysplastic hamster check pouch with non-acid-disassembling Au NCs. Images of DMBA-applied, early-stage dysplastic hamster cheek pouches, obtained by SD-OCT (**a**) and DvOCT (**b**) after administering non-acid-disassembling Au NCs. Scale bar: 100 µm. The images were taken before (base) and 20 and 40 min after administering non-acid-disassembling Au NCs. The color-coded Doppler variance in DvOCT images of early-stage dysplasia (5 dotted regions) was quantified and compared with that of normal tissue (**c**) (yellow area: epithelial layers)

In contrast, the Doppler variance in epithelial layers in the dysplastic tissue was lower than that in the normal tissue, when non-acid-disassembling Au NCs (control Au NCs) were administered (Fig. 9c). In general, contrast of OCT and DvOCT images in the dysplastic tissue were enhanced by applying non-acid-degradable Au NCs (Figs. 8, 9a, b), indicating stimuli-independent optical contrast enhancement by both types of Au NCs. However, the scattering signal changes in the dysplastic tissue treated with non-acid-disassembling Au NCs, in comparison with normal tissue (Additional file 1: Figures S3 and S4), was smaller than when acid-disassembling Au NCs were administered. Moreover, higher Doppler variance in the epithelial layers in the dysplastic tissue compared with the normal tissue was found only when acid-disassembling Au NCs were administered (Figs. 7, 9). Therefore, the results shown in Figs. 7 and 9 clearly demonstrate the detection of early-stage dysplasia with pinpoint accuracy, which is challenging to confirm with most imaging modalities, by obtaining stimuli-triggered, multimodal (scattering and Doppler variance) optical signal changes using novel nanoparticle-based optical contrast agents.

## Conclusions

This study demonstrates the high feasibility of identifying neoplastic change by employing stimuli-responsive contrast agents for a clinically relevant optical imaging system (i.e., optical coherence tomography). A clustered form of Au NPs (i.e., Au NCs) exhibited enhanced scattering and slow Brownian motion in normal tissue, while Au NCs reverted to individual Au NPs and showed diminished scattering and fast Brownian motion upon acid-hydrolysis of the clustering linkers in mildly acidic early-stage dysplastic tissue. This study’s hypothesis of obtaining multiple, stimuli-responsive optical signal changes using molecularly engineered inorganic NPs can further be expanded to drug delivery and other imaging applications. Stimuli-transforming clusters can be synthesized using a variety of nanomaterials and tuned to be responsive to specific pathological stimuli (e.g., hyperthermia, enzymes, and hypoxia) as well as external triggers (e.g., magnetic force, electric field, and light). Combined incorporation of therapeutic and diagnostic (imaging) nanomaterials can be used to develop novel theranostic agents.

## Additional file

**Additional file 1.** Additional figures and tables.
