# Supplementary material for: Stimuli-disassembling gold nanoclusters for diagnosis of early stage oral cancer by optical coherence tomography
Source: Nano Converg. 2018 Jan 26;5:3. doi: 10.1186/s40580-018-0134-5 (PMC5785591; doi:10.1186/s40580-018-0134-5)
Supplement: Supplementary file 1 — Additional file 1. Additional figures and tables. [file 40580_2018_134_MOESM1_ESM.docx]

Supporting information

**Stimuli-Disassembling Gold Nanoclusters for Diagnosis of Early Stage Oral Cancer by Optical Coherence Tomography**

Chang Soo Kim,^a, b^ Dominique Ingato,^a^ Petra Wilder-Smith,^b^ Zhongping Chen ,^a,b,c,*^ Young Jik Kwon ^a,c,d,e,*^

a University of California, Department of Chemical Engineering and Materials Science, 916 Engineering Tower, Irvine, California 92697-2575, USA;

b University of California, Beckman Laser Institute, 1002 Health Sciences Road East, Irvine, California 92612, USA;

c University of California, Department of Biomedical Engineering, 3120 Natural Sciences II, Irvine, CA 92697-2715, USA;

d University of California, Department of Pharmaceutical Sciences, 147 Bison Modular, Irvine, California 92697, USA;

e University of California, Department of Molecular Biology and Biochemistry, 3205 McGaugh Hall, Irvine, CA 92697-3900, USA

**DA**

**A**

**CA**

**B**


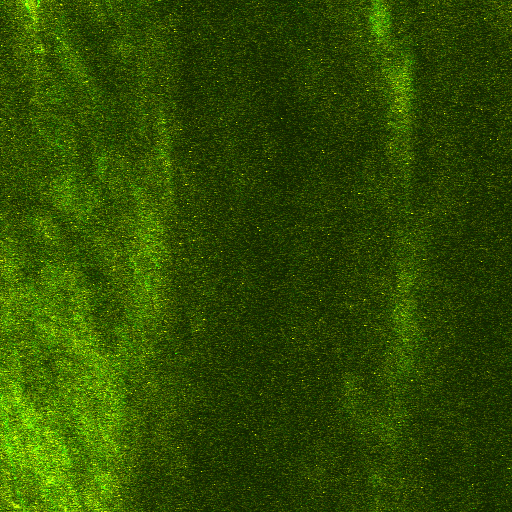

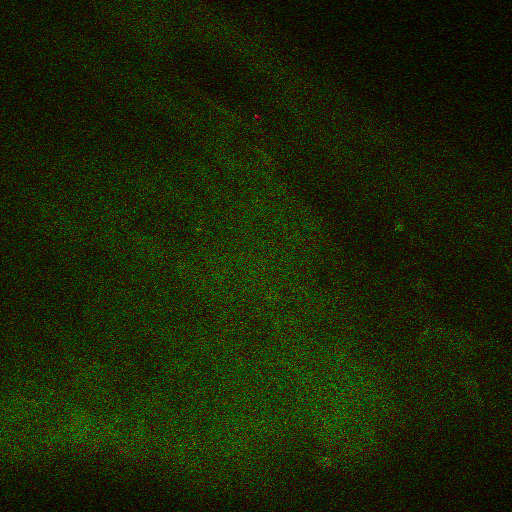

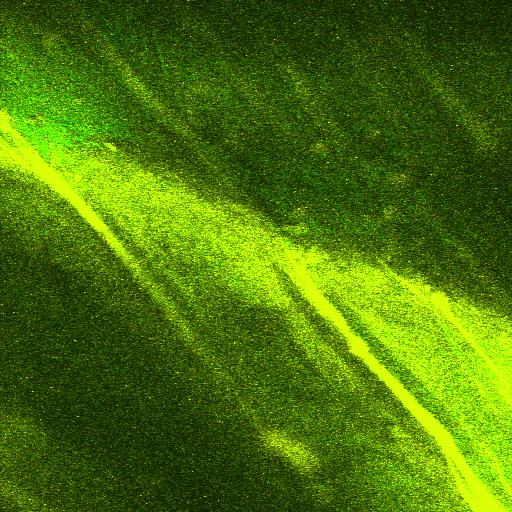

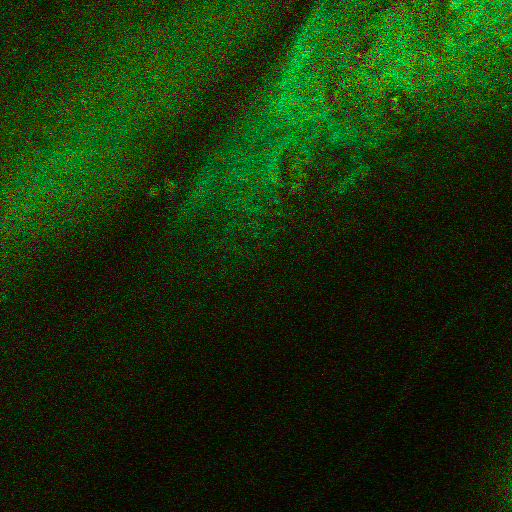


**Figure S1. Confocal images of dysplastic hamster cheek pouch with applied SNARF-conjugated Au NCs.** Confocal images of dysplastic tissue were obtained before (a) and after (b) SNARF Au NC administration. Confocal images of normal tissue were obtained before (c) and after (d) SNARF Au NC administration. Scale bar: 100 μm.


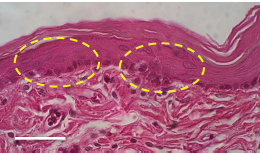


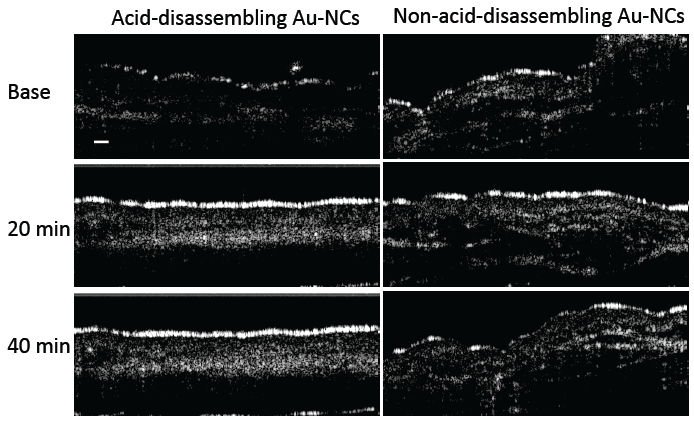
**Figure S2. Dotted circles indicate thickened epithelial layers.** Dotted circles indicate thickened epithelial layers indicating early-stage dysplasia. Scale bar: 100 μm.

Acid-disassembling AuNCs Non-acid-disassembling AuNCs

**Acid-disassembling Au NCs Non-acid-disassembling Au NCs**

**Figure S3. OCT images of normal hamster cheek pouches.** The images were obtained by an SD-OCT system. The images were taken before (base) and 20 and 40 min after administering acid-disassembling and non-acid disassembling AuNCs. Scale bar: 100 µm.

**Acid-disassembling Au NCs Non-acid-disassembling Au NCs**


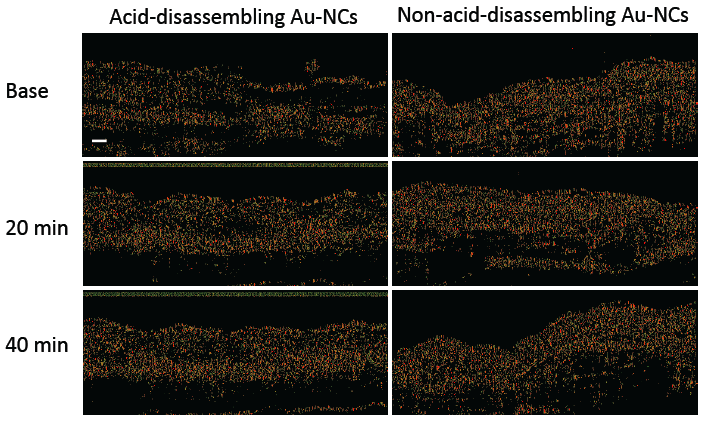


**Figure S4. DvOCT images of normal hamster cheek pouches.** The images were obtained by an SD-OCT system. The images were taken before (base) and 20 and 40 min after administering acid-disassembling and non-acid disassembling Au-NCs. Scale bar: 100 µm.

***
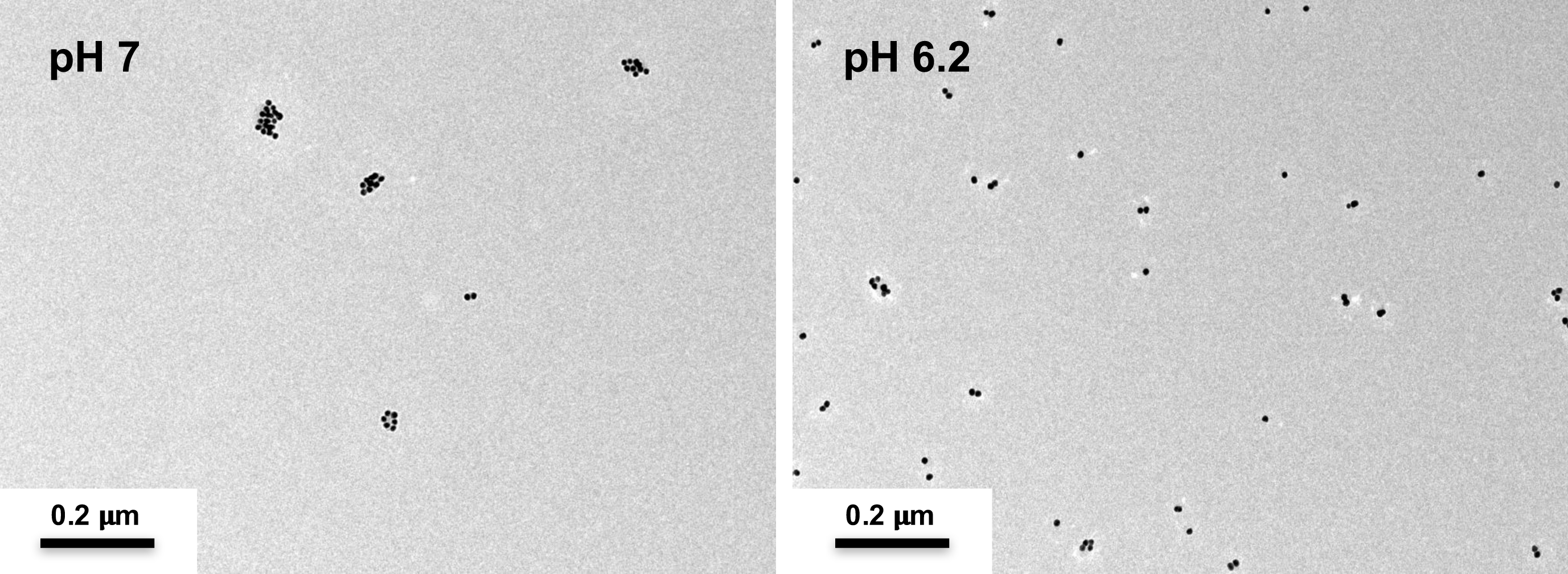
***

**Figure S5. TEM images of Au NCs.** Acid-degradable Au NCs incubated at pH 7 and pH 6.2.

**DI H_2_O pH 5.0**

**Figure S6. Intensity quantification for Figure 4A and 4B** (*p <* 0.0001).

***
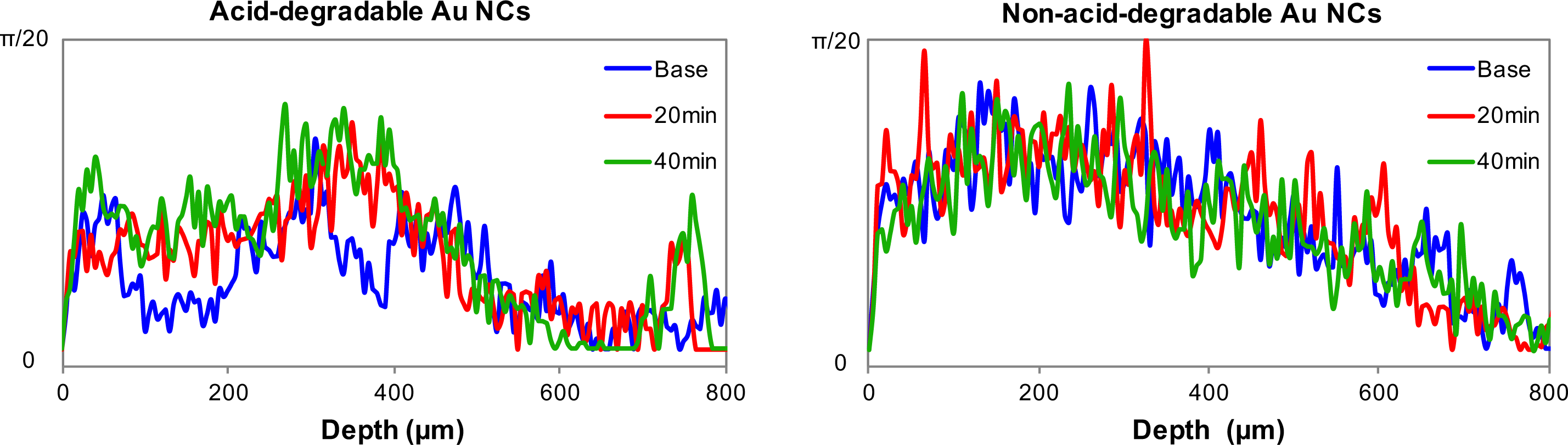
***

**Figure S7. Normal tissue with acid-degradable and non-acid degradable Au NCs.** The color-coded Doppler variance in DvOCT images of normal tissue.

**Table S1. Quantification of OCT results**

A. OCT signal for Acid-degradable Au-NCs

|  | Normal | STDEV | Dysplasia | STDEV |
| --- | --- | --- | --- | --- |
| Base | 3.22 | 1.30 | 1.52 | 1.16 |
| 20min | 18.34 | 3.74 | 10.77 | 4.08 |
| 40min | 20.68 | 2.94 | 12.68 | 4.67 |

B. OCT signal for Non-acid-degradable Au-NCs

|  | Normal | STDEV | Dysplasia | STDEV |
| --- | --- | --- | --- | --- |
| Base | 9.37 | 3.33 | 4.59 | 2.65 |
| 20min | 14.55 | 3.50 | 13.94 | 5.31 |
| 40min | 10.57 | 5.50 | 15.24 | 4.28 |

C. Signal improvement for Acid-degradable Au-NCs

|  | Normal | Dysplasia |
| --- | --- | --- |
| 20 min/ Base | 5.69 | 7.08 |
| 40 min/ Base | 6.42 | 8.34 |

D. Signal improvement for Non-acid-degradable Au-NCs

|  | Normal | Dysplasia |
| --- | --- | --- |
| 20 min/ Base | 1.55 | 3.04 |
| 40 min/ Base | 1.13 | 3.32 |

**Table S2. *p* values for Figure 7 and 9 between normal and dysplastic tissue**

|  | Acid-degradable AuNCs | Non-acid-degradable AuNCs |
| --- | --- | --- |
| Base | 7.74859E-09 | 1.15858E-24 |
| 20 min | 2.30387E-05 | 0.009715547 |
| 40 min | 0.001525574 | 0.048017943 |
